# Enhancement of Chlorogenic Acid Production in Hairy Roots of *Platycodon grandiflorum* by Over-Expression of An *Arabidopsis thaliana* Transcription Factor *AtPAP1*

**DOI:** 10.3390/ijms150814743

**Published:** 2014-08-22

**Authors:** Pham Anh Tuan, Do Yeon Kwon, Sanghyun Lee, Mariadhas Valan Arasu, Naif Abdullah Al-Dhabi, Nam Il Park, Sang Un Park

**Affiliations:** 1 Department of Crop Science, Chungnam National University, 99 Daehak-ro, Yuseong-Gu, Daejeon 305-764, Korea; E-Mails: tuan_pham_6885@yahoo.com (P.A.T.); kdy309@naver.com (D.Y.K.); 2 Department of Integrative Plant Science, Chung-Ang University, Anseong 456-756, Korea; E-Mail: slee@cau.ac.kr; 3 Department of Botany and Microbiology, Addiriyah Chair for Environmental Studies, College of Science, King Saud University, PO Box 2455, Riyadh 11451, Saudi Arabia; E-Mails: mvalanarasu@gmail.com (M.V.A.); naldhabi@ksu.edu.sa (N.A.A.-D.); 4 Department of Plant Science, Gangneung-Wonju National University, 7 Jukheon-gil, Gangneung-si, Gangwon-do 210-702, Korea; 5 Visiting Professor Program (VPP), King Saud University, PO Box 2455, Riyadh 11451, Saudi Arabia

**Keywords:** *Agrobacterium rhizogenes*, chlorogenic acid, gene over-expression, hairy root, *Platycodon grandiflorum*, transcription factor, production of anthocyanin pigment 1

## Abstract

To improve the production of chlorogenic acid (CGA) in hairy roots of *Platycodon grandiflorum*, we induced over-expression of *Arabidopsis thaliana* transcription factor production of anthocyanin pigment (AtPAP1) using an *Agrobacterium rhizogenes*-mediated transformation system. Twelve hairy root lines showing over-expression of *AtPAP1* were generated. In order to investigate the regulation of *AtPAP1* on the activities of CGA biosynthetic genes, the expression levels of seven *P. grandiflorum* CGA biosynthetic genes were analyzed in the hairy root line that had the greatest accumulation of *AtPAP1* transcript, OxPAP1-1. The introduction of AtPAP1 increased the mRNA levels of all examined CGA biosynthetic genes and resulted in a 900% up-regulation of CGA accumulation in OxPAP1-1 hairy roots relative to controls. This suggests that *P. grandiflorum* hairy roots that over-express the *AtPAP1* gene are a potential alternative source of roots for the production of CGA.

## 1. Introduction

**Figure 1:**
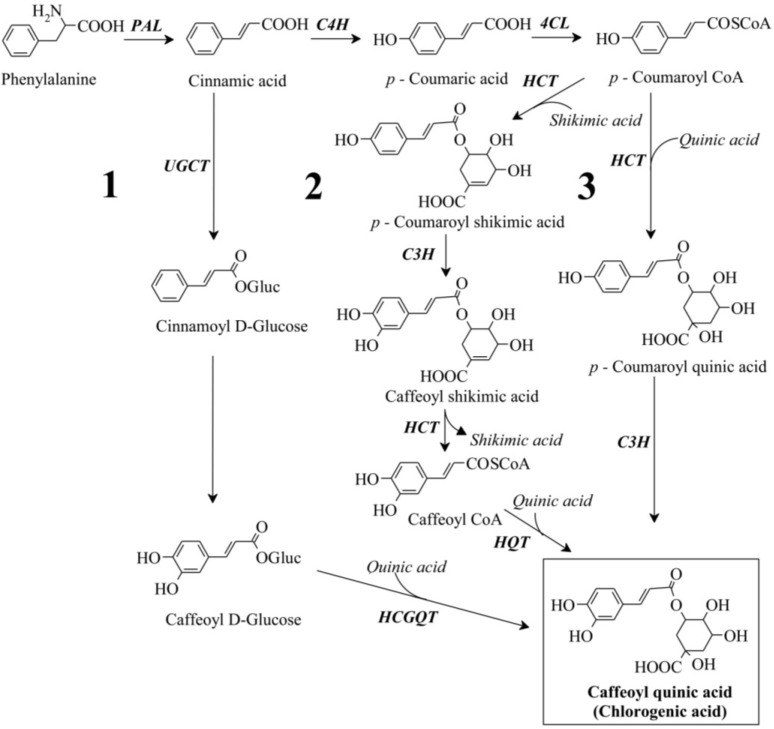
Three proposed pathways for chlorogenic acid synthesis in plants (labeled **1**, **2** and **3**). PAL, phenylalanine ammonia lyase; C4H, cinnamate 4-hydroxylase; 4CL, 4-coumarate CoA ligase; HCT, hydroxycinnamoyl CoA shikimate/quinate hydroxycinnamoyl transferase; C3H, *p-*coumarate 3'-hydroxylase; HQT, hydroxycinnamoyl CoA quinate hydroxycinnamoyl transferase; UGCT, UDP glucose: cinnamate glucosyl transferase; HCGQT, hydroxycinnamoyl d-glucose: quinate hydroxycinnamoyl transferase.

Chlorogenic acid (CGA), which is composed of a family of esters between certain trans-cinnamic acids and quinic acid, is the major soluble phenolic compound found in tomatoes, potatoes, and tobacco. CGA is an important for plant metabolism. For example, it is a precursor of G- and S-type lignin biosynthesis and plays a prominent role in tissue senescence [1]. CGA possesses diverse health effects; it performs well as an antioxidant, particularly in protecting lipids from peroxidation, the initial event in atherosclerosis [2]. Although CGA was identified decades ago, the complete pathway for the synthesis of CGA is still debated [3]. Three distinct pathways have been considered (Figure 1): (1) caffeoyl-glycoside is used as the activated intermediate, (2) synthesis of CGA from caffeoyl-CoA and quinic acid by hydroxycinnamoyl CoA quinate hydroxycinnamoyl transferase (HQT), and (3) hydroxylation of *p-*coumaroyl-quiric acid by *p-*coumarate 3'-hydroxylase (C3H) to form CGA [4,5,6]. Due to the importance of CGA for plant and human health, the metabolic engineering of CGA biosynthesis in plants has attracted the interest of researchers for long time. Over-expression of transcription factors that regulate the transcription of genes involved in the phenylpropanoid pathway is an effective tool for engineering high levels of CGA. *Arabidopsis thaliana* production of anthocyanin pigment (AtPAP1) significantly increases accumulation of CGA [7] and activates most of the phenylpropanoid pathway genes in plants [8,9].

The root of *Platycodon grandiflorum* have been reported to have a wide range of health benefits, including anti-inflammatory, anti-lipidemic, anti-hypercholesteroleamic, and anti-obesity effects [10,11]. Many beneficial compounds have been isolated from the roots of *P. grandiflorum*, such as saponins, platycodon, phytosterols, polyacetylenes, and phenylpropanoids [12,13]. On the other hand, hairy roots, which are induced at or near the site of infection of a plant with *Agrobacterium rhizogenes*, have recently been considered as a promising alternative material for large-scale production of secondary metabolites because of their fast growth rate and biochemical and phenotypic stability [14]. Given the good characteristics of hairy roots and the richness of beneficial compounds in *P. grandiflorum*, the hairy roots of *P. grandiflorum* are probably a good model for assessing improvements in the production of a natural product through biotechnology.

The aim of this study was to establish an efficient approach for improvement of CGA production in hairy roots of *P. grandiflorum* by *A. rhizogenes*-mediated co-transformation. Here, the effect of exogenous AtPAP1 over-expression on the expression levels of CGA biosynthetic genes and CGA accumulation was investigated in *P. grandiflorum* hairy roots.

## 2. Results and Discussion

### 2.1. Establishment of Transgenic Hairy Root Lines

**Figure 2:**
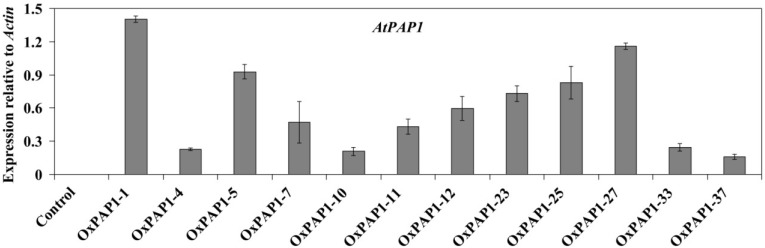
Expression levels of the *Arabidopsis thaliana* transcription factor production of anthocyanin pigment (*AtPAP1*) gene in *P. grandiflorum* transgenic hairy root lines. The height of each bar and the error bars show the mean and standard error, respectively, of three independent measurements. Control and OxPAP1-*n*, *P. grandiflorum* transgenic hairy roots by *A. rhizogenes* strain R1000 with pK7FWG2 plant binary expression vector carrying GUS and AtPAP1, respectively (*n* indicates the line number).

**Figure 3:**
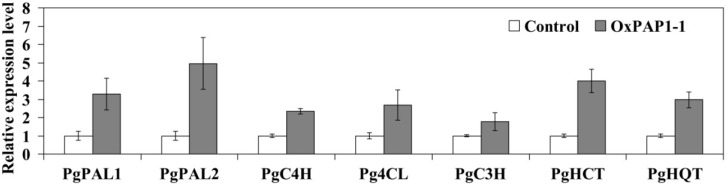
Expression levels of *PgPAL1*, *PgPAL2*, *PgC4H*, *Pg4CL*, *PgC3H*, *PgHCT*, and *PgHQT* in the *P. grandiflorum* OxPAP1-1 hairy root line. Control hairy roots were assigned a value of 1 and results are expressed as means and standard deviation relative to the controls.

The over-expression of AtPAP1 driven by the CaMV 35S promoter was induced in hairy roots of *P. grandiflorum* using *A. rhizogenes* strain R1000. The transgenic hairy roots over-expressing AtPAP1 were not morphologically different and did not differ in growth rate from control hairy roots that had CaMV 35S promoter-GUS fusion. Twelve hairy root lines showing over-expression of *AtPAP1* were generated, and no *AtPAP1* expression was detected in the control *P. grandiflorum* hairy root line (Figure 2). Of the transgenic hairy root lines, the OxPAP1-1 line had the greatest accumulation of *AtPAP1*transcripts and was selected for further analysis. In order to investigate the regulation of CGA biosynthetic genes by *AtPAP1*, the expression levels of *PgPAL1*, *PgPAL2*, *PgC4H*, *Pg4CL*, *PgC3H*, *PgHCT*, and *PgHQT* were analyzed in the OxPAP1-1 line. As seen in Figure 3, the introduction of the *AtPAP1* gene dramatically increased the expression levels of all examined CGA biosynthetic genes in *P. grandiflorum* hairy roots. Control hairy roots were assigned a value of 1, and the expression levels of seven *P. grandiflorum* genes involved in CGA biosynthesis were several-fold higher in the OxPAP1-1 line than in control hairy roots. Relative to controls, the greatest increase in mRNA levels were observed for PgPAL2, PgHCT, and PgPAL1, with fold changes of 4.95, 3.99, and 3.29, respectively.

### 2.2. Chlorogenic Acid Production in Hairy Roots Over-Expressing AtPAP1

**Figure 4:**
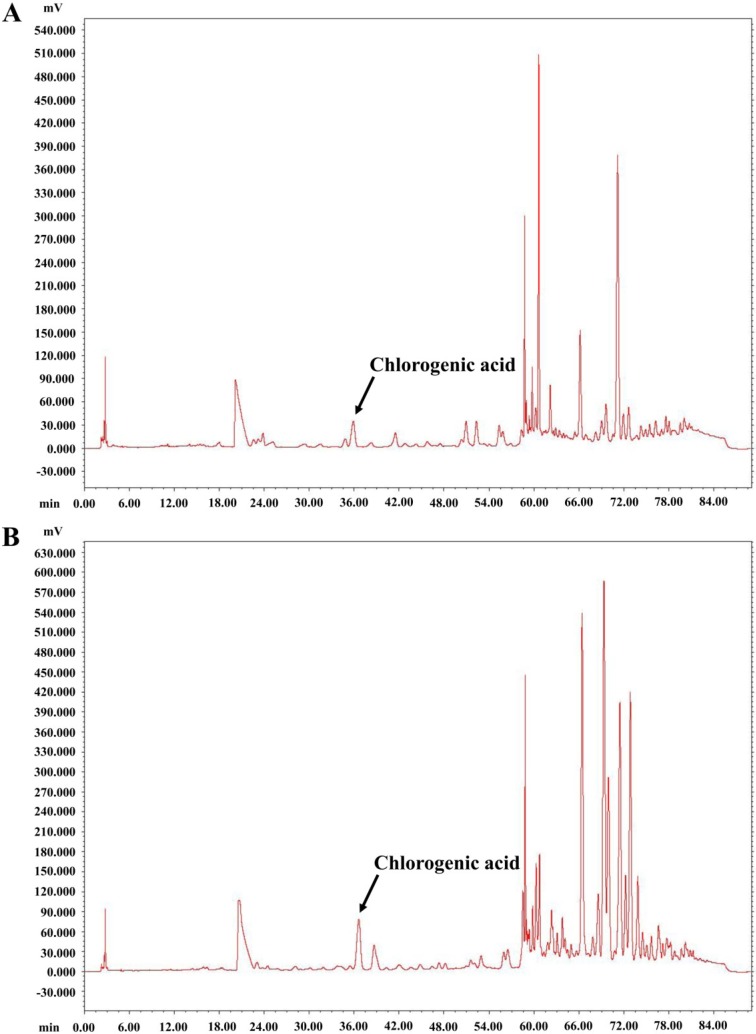
High-Performance Liquid Chromatography (HPLC) chromatograms of chlorogenic acid (CGA) analysis from control hairy roots (**A**) and OxPAP1-1 hairy roots (**B**) of *P. grandiflorum*.

**Figure 5:**
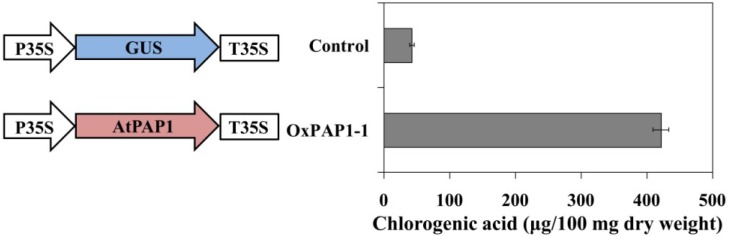
Chlorogenic acid content in *P. grandiflorum* hairy roots transformed with AtPAP1 over-expression constructs. The height of each bar and the error bars show the mean and standard error, respectively, of three independent measurements. P35S, CaMV 35S promoter; T35S, 3' termination signal of CaMV 35S gene.

HPLC analysis showed that, relative to control hairy roots, CGA accumulation significantly increased in transgenic hairy roots transformed with *A*. * thaliana* transcription factor PAP1 (Figure 4 and Figure 5). The CGA content in the OxPAP1-1 hairy root line was 421.31 μg/100 mg of dry weight, *i.e.*, 9.89-time higher than that the control hairy root line (42.60 μg/100 mg).

Introduction of individual biosynthetic genes to transgenic plants increases the accumulation of CGA up to several times that of wild-type plants. CGA levels are increased approximately 3-fold by overexpression of PAL in transgenic tobacco [15]. Gene-silencing of *HQT* reduces 98% of CGA, while over-expression increases CGA content up to 85% in transformed tomato leaves [4]. The over-expression of transcription factors, which stimulate the expression levels of almost all of the biosynthetic genes, can further increase the CGA content in tomato (22 times) [16] and *Saussurea involucrate* (15 times) [7]. In this study, we found that the introduction of AtPAP1 led to increased mRNA levels for all examined CGA biosynthetic genes and a 9.89-time increase of CGA accumulation in *P. grandiflorum* hairy roots. Over-expression of AtPAP1 not only stimulated the expression levels of down-stream genes in the CGA biosynthetic pathway, causing the induction of CGA accumulation, but also increased the activities of four genes involved in the general phenylpropanoid pathway. The general phenylpropanoid pathway generates an enormous array of secondary metabolites, such as flavonoids, stilbenes, lignins, and rutin [17]. This suggests that the induction of PgPAP1, PgPAL2, PgC4H, and Pg4CL in the OxPAP1-1 line also up-regulated the biosynthesis of other phenylpropanoid compounds. Further studies of other compounds in *P. grandiflorum* hairy roots are needed to clarify this suggestion. In addition, the expression profiles of PgPAL1, PgPAL2, PgC4H, and Pg4CL in OxPAP1-1 hairy roots will enhance our understanding of the transcriptional regulation of phenylpropanoid biosynthesis in *P. grandiflorum*.

## 3. Experimental Section

### 3.1. Plasmid Construction

Polymerase chain reaction (PCR) was used to generate *AtPAP1* coding regions (GenBank accession number: NM_104541.3). Purified DNA fragments of eight genes were recombined into a pDONR 221 vector using BP clonase II (Invitrogen, Carlsbad, CA, USA) according to the manufacturer’s instructions. Recombinant plasmids were transformed into TOP10 *Escherichia coli* cells (Invitrogen, Carlsbad, CA, USA) using kanamycin selection at a final concentration of 50 g/mL. After confirming the plasmid inserts by sequencing, the *pDONR221* gene construct was excised and recombined into pK7FWG2 (http://www.psb.ugent.be/gateway/) [18] using LR clonase (Invitrogen, Carlsbad, CA, USA). The over-expression construct consisted of a cauliflower mosaic virus (CaMV) 35S promoter, AtPAP1 sequences, and the neomycin phosphotransferase (*NPTII*) gene as a selectable marker (Figure 5). The pK7FWG2 plasmids were transferred into *A. rhizogenes* strain R1000 by electroporation and grown at 28 °C on Luria-Bertani medium with kanamycin at a final concentration of 50 mg/L.

### 3.2. Establishment of Hairy Root Transformation

Excised cotyledons of *P. grandiflorum* from 12-day-old seedlings were used as explants for co-cultivation with *A. rhizogenes* strain R1000 that harbored AtPAP1 over-expression constructs. A similar pK7FWG2 vector with a CaMV 35S promoter-GUS fusion and the *NPTII* gene as a selectable marker was used as a control. The transformation of *P. grandiflorum* using *A. rhizogenes* was carried out as previously described by our group [19]. Hairy roots that were induced on plates containing 75 mg/L of kanamycin were examined by PCR with primers for the *NPTII* (kanamycin resistance) gene and *rol* genes (data not shown). Approximately 100 mg of different hairy root lines that had confirmed integration of over-expression were transferred to 30 mL of 1/2 Murashige and Skoog liquid medium in 100 mL flasks. Hairy root cultures were maintained at 25 °C on a gyratory shaker (100 rpm) in a growth chamber. Light was provided by standard cool white fluorescent tubes with a flux rate of 35 µmol s^−1^ m^−2^ and a 16 h photoperiod. After 3 weeks, hairy root samples were frozen in liquid nitrogen immediately after harvesting and then stored at −80 °C and/or freeze-dried for total RNA isolation and/or HPLC analysis.

### 3.3. RNA Isolation and cDNA Synthesis

Hairy root samples were ground into powder in a mortar with liquid nitrogen, and total RNA was isolated using a Plant Total RNA Mini kit (Geneaid, Sijhih, Taiwan) according to the manufacturer’s instructions. The quality and concentration of total extracted RNA was determined by 1% agarose gel electrophoresis and spectrophotometric analysis, respectively. For quantitative real-time PCR, 1 μg of total RNA was used for reverse transcription using the ReverTra Ace-R kit (Toyobo, Osaka, Japan), and a 20-fold dilution of the resulting 20 μL cDNA was used as template.

### 3.4. Quantitative Real-time PCR Analysis

Real-time PCR primers were designed using the Primer3 website [20] (http://frodo.wi.mit.edu/primer3/) and were based on the *P. grandiflorum* phenylalanine ammonia lyase (PAL1 and PAL2), cinnamate 4-hydroxylase (C4H), 4-coumarate CoA ligase (4CL), C3H, HCT, and HQT sequences, which were obtained as part of another study (unpublished sequences) [21], and AtPAP1 (Table 1). Real-time PCR products were tested for specificity of fragment sizes, melting curves, and sequences by PCR, real-time PCR, and cloning into a T-Blunt vector for sequencing, respectively. The expression of genes was calculated by relative quantification using the *P. grandiflorum* actin housekeeping gene (JF781303) as the reference. To quantify the standard, PCR products amplified from cDNA were purified, and the concentration of the products was measured in order to calculate the number of cDNA copies. Real-time PCR reactions were carried out in a 20 μL reaction mix containing 5 μL of template cDNA, 10 μL of 1× SYBR Green Real-time PCR Master Mix (Toyobo, Osaka, Japan), 0.5 μL of each primer (10 μM), and diethylpyrocarbonate water. Thermal cycling conditions were as follows: 95 °C for 5 min; 40 cycles of 95 °C for 15 s, 56 °C for 15 s, and 72 °C for 20 s. Each run contained a series of standards and a negative control (containing water instead of cDNA). PCR products were analyzed using Bio-Rad CFX Manager 2.0 software (Bio-Rad Laboratories, Hercules, CA, USA). Three replications for each sample were used in the real-time analyses.

**Table 1:** Primers used for quantitative real-time PCR.

Primer	Sequence (5' to 3')	Amplicon (Base Pairs)
PgPAL1_F	CCACAACGTCACCCCTGTTT	140
PgPAL1_R	CATTAAGGACTTGCCCGGTT	
PgPAL2_F	AACCATAACATTACCCCATGCC	146
PgPAL2_R	GCAGCAGTAAGTGATTGTCCATCA	
PgC4H_F	CCTTTTGGTCCCTCACATGAAC	175
PgC4H_R	AGCCTCGACCTTGGACTCTTCT	
Pg4CL_F	CTTTGCCAAGGAACCATACGAG	183
Pg4CL_R	CTCTCAGTGGCCTCTGGATCAT	
PgC3H_F	CGATTATGGGCCTCATTATGTTA	162
PgC3H_R	CTCTTTCCCTCGTTATCAGGATT	
PgHCT_F	GGCTCGTGGACTCGATGTCA	150
PgHCT_R	GGACTGATTGAGCATTTGCAGG	
PgHQT_F	GAACGCCTCCGTTCAAAACA	160
PgHQT_R	GCGGCCAAGATCACGTAACTA	
PgActin_F	CCATACAGTCCCCATTTATGAAG	170
PgActin_R	GCTAACTTCTCCTTCATGTCTCTCA	
AtPAP1_F	AATGGCACCAAGTTCCTGTAAGA	141
AtPAP1_R	TATGAAGGCGAAGAAGAAGATCG	

### 3.5. High-Performance Liquid Chromatography (HPLC) Analysis

The external standards for 3-(3,4-dihydroxycinnamoyl) quinic acid (CGA), were purchased from Extrasynthese (Genay, France). HPLC-grade methanol (MeOH) was purchased from Wako Pure Chemical Industries (Osaka, Japan). Formic acid and acetic acid were provided by Kanto Chemical Co., Inc. (Tokyo, Japan) and Junsei Chemical Co., Ltd. (Kyoto, Japan), respectively. For HPLC analysis, sample extracts were filtered through a 0.45 μm poly filter and then diluted 2-fold with MeOH prior to analysis. Samples were determined using a Futecs model NS-4000 HPLC apparatus (Daejeon, Korea) with a UV–vis detector and an auto sampler. The analysis was monitored at 280 nm and performed using a C_18_ column (250 mm × 4.6 mm × 5 μm; RStech, Daejeon, Korea); the mobile phase was a gradient prepared from mixtures of 0.15% acetic acid and methanol, and the column was maintained at 30 °C. The flow rate was set at 1.0 mL/min, and the injection volume was 20 μL. Quantification of CGA was based on peak areas and calculated as equivalents of representative standard compounds. All quantities are expressed as μg/100 mg of dry weight, and all samples were analyzed in triplicate.

## 4. Conclusions

This study demonstrates the successful metabolic engineering of CGA biosynthesis in hairy roots of *P. grandiflorum* through genetic modification with AtPAP1 using *A. rhizogenes*. Over-expression constructs of AtPAP1 are an effective method for enriching the formation of CGA. The establishment of transgenic hairy roots with rich CGA accumulation represents a novel and potential alternative source of *P. grandiflorum* roots for medicinal and commercial applications.
